# Adulterants present in the San Diego county fentanyl supply: a laboratory analysis of seized law enforcement samples

**DOI:** 10.1186/s12889-024-18459-0

**Published:** 2024-03-29

**Authors:** Henrik Galust, Justin A. Seltzer, Jeremy R. Hardin, Nathan A. Friedman, Jeff Salamat, Richard F. Clark, Jennifer Harmon

**Affiliations:** 1 Division of Medical Toxicology, Department of Emergency Medicine, UC San Diego Health, 200 W. Arbor Dr. #8676, 92103 San Diego, CA USA; 2 VA San Diego Healthcare System, San Diego, CA USA; 3 California Poison Control System, San Diego, CA USA; 4 San Diego County Sheriff’s Crime Laboratory. John F. Duffy Administrative Center, 9621. Ridgehaven Ct, 92123 San Diego, CA USA

**Keywords:** Adulterant, Contaminant, Opioid, Fentanyl, Drug abuse, Xylazine, Pharmacosurveillance, Pharmacovigilance, Forensic toxicology, Toxicology

## Abstract

**Background:**

The opioid overdose crisis is one of the worst public health crises ever to face the US and emerging evidence suggests its effects are compounded by the presence of drug adulterants. Here we report our efforts to characterize the adulterants present within the local fentanyl supply of San Diego County, obtained from undifferentiated drug samples seized by local law enforcement over the calendar year 2021.

**Methods:**

Thirty-two participating local law enforcement agencies across San Diego submitted 4838 unknown individual illicit drug samples (total of 312 kg) to the San Diego County Sheriff’s Department Regional Crime Laboratory for identification.

**Results:**

Qualitative analysis of these samples via FTIR and GC-MS identified methamphetamine (38.7%), fentanyl (20.8%), diacetylmorphine (heroin) (10.2%), codeine (5.8%) and alprazolam (4.3%) as the most common illicit substances and the presence of 52 unique adulterants. The most common adulterants included 4-methylaminoantipyrine (4-MAAP) (10.9%), mannitol (9%), acetaminophen (8.5%), methamphetamine (4.2%), diacetylmorphine (heroin) (3.6%), tramadol (1.9%), and xylazine (1.7%). Several additional pharmacologically active adulterants and contaminants of interest were also identified.

**Conclusion:**

This analysis is vital for public health use and harm reduction efforts at the level of the individual consumer. Continued direct surveillance of the drug supply is necessary for the detection of potentially harmful adulterants that may pose serious threats to the public.

## Background

According to a nationwide survey conducted in 2020, 6.6% of individuals over the age of 12 reported at least one substance use disorder of an illicit drug of abuse in the prior year [1]. In the same year, more than 105,000 deaths were associated with drug overdoses; nearly 75% of these were attributed to illicit opioid use [2]. Within this context, there is an ongoing public health concern related to the presence of “adulterants” and “contaminants” within drug supplies [3–5].

Adulterants are pharmacologically active or inactive ingredients added to increase bulk as a cost-saving measure, produce synergistic drug effects to enhance or improve the effects of the diluted drug, help enhance drug absorption, or reduce the amount of drug necessary to achieve the desired effect [6, 7]. Potentially harmful adulterants have historically been associated with morbidity and mortality [8–13]. On the other hand, contaminants are unintentional and have no role in augmenting the intended drug. They are commonly precursors or by-products of manufacturing or storage, remnants of low-quality manufacturing techniques and storage practices [6]. The landscape of drug adulterants specifically is complex and rapidly evolving, often subject to various economic and law-enforcement pressures experienced by illicit drug manufacturers. Consequently, routine public health surveillance of the drug supply is necessary [6].

Here we report our efforts to characterize the adulterants present within our local fentanyl supply in San Diego County. By analyzing seized fentanyl samples, we aim to provide insight into current adulteration trends and raise awareness of potentially harmful and clinically relevant adulterants in the drug supply.

## Methods

From January 4, 2021 to December 30, 2022, 32 participating local law enforcement agencies across our county submitted 4838 unknown individual illicit drug samples (total of 312 kg) to the County Sheriff’s Department Regional Crime Laboratory for identification.

Non-targeted analysis was employed to identify controlled substances using identification criteria. Seized drug samples were initially analyzed using presumptive colorimetric testing and confirmed using FTIR and/or GCMS. FTIR was performed using a Thermo Scientific Nicolet iS10 infrared spectrometer with a Specac Golden Gate Attenuated Total Reflectance (ATR) attachment. The analyzed sample was directly placed onto a diamond crystal for analysis and pressure was applied via a sapphire-fitted anvil to create a uniform spread on the diamond crystal. The FTIR optical bench collected 32 scans at a resolution of 4.000 cm-1 with a sample gain of 8.0 and optical velocity of 0.6329 with the aperture set to 80.00. Omnic (Thermo Scientific: Version 8.3) was used for acquisition and analysis. The analyte spectrum was compared to our own created and verified reference spectrum library. GCMS libraries included SWGDRUG’s MS library, Wiley12/NIST11 MS library, Wiley’s Designer Drugs MS library (2021, 2022), Wiley’s MPW MS library, Cayman Chemical’s MS library, and the San Diego Sheriff’s Department in-house crime laboratory library.

Gas chromatography–mass spectrometry (GC-MS) is routinely employed in crime laboratories for the analysis of drugs of abuse [14]. The samples were prepared by dissolving approximately 1 mg of analyte in a suitable solvent (ethanol, methanol, chloroform, methylene chloride, or other suitable solvent). Liquid-liquid extractions were used when necessary and analyzed as filtrates. Proadifen was added to 1.5 mL of the extract as an internal reference at a concentration of approximately 1 mg/mL in a sample vial for monitoring instrument conditions. 1 µL was injected onto an Agilent 7890 Gas Chromatograph fitted with a crossbonded diphenyl dimethyl polysiloxane (5%) column (e.g. Restek Rxi-5Sil MS). The injection is generally on pulsed spitless mode with a pulse pressure of 50 PSI and held for 30 s. Column length was 15 m, with an internal diameter of 0.25 mm and film thickness of 0.25 μm. General method parameters started at 80 °C and increased up to 280 °C at a rate of 20 °C per minute with a 12-minute run time. Eluents were introduced into an Agilent 5977 mass spectrometer set to positive ion polarity. Fragmentation was by electron impact ionization at 70 eV. The MS was operated in full scan with a mass window of 25 to 600 m/z at a threshold of 150 with a scan speed of 781.

## Results

**Fig. 1:**
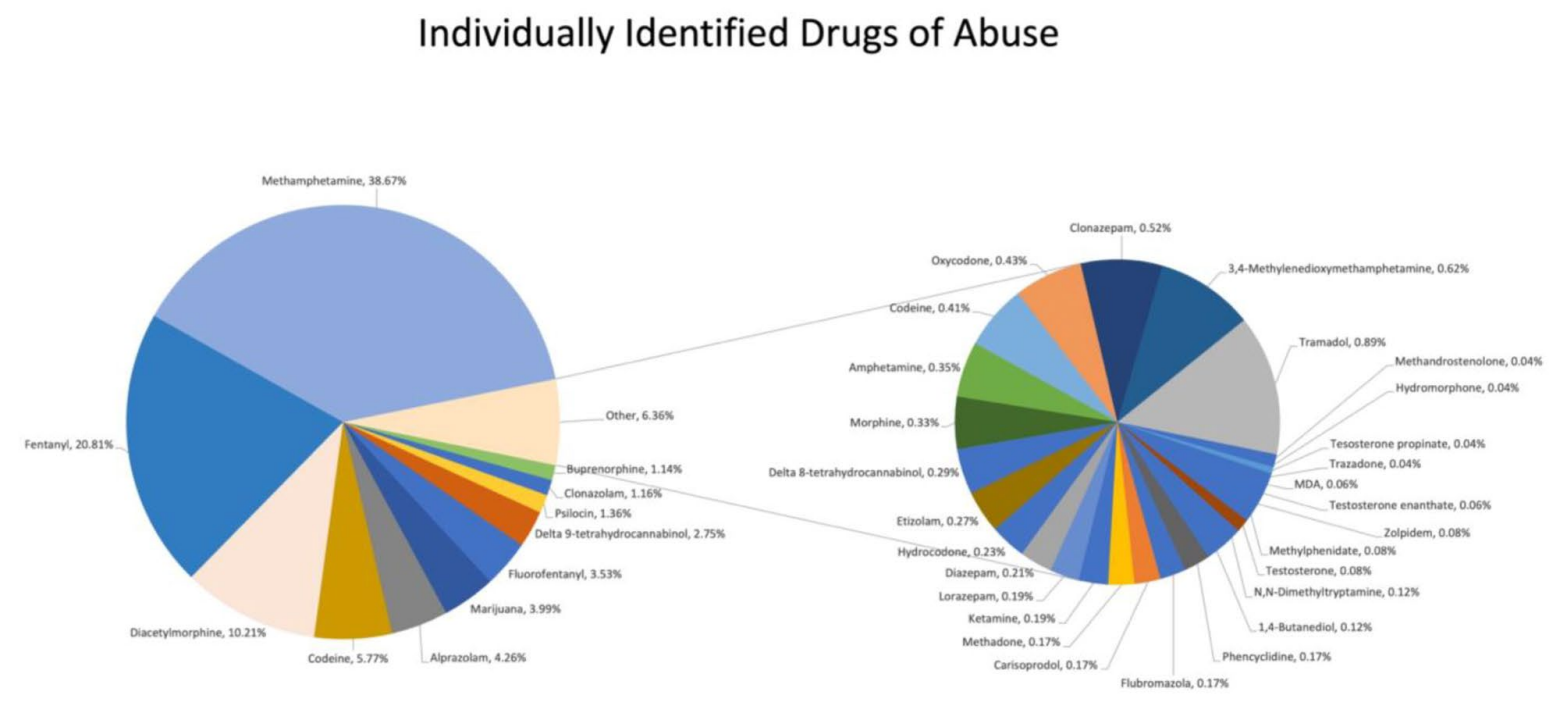
39 individual drugs of abuse identified from 4838 submitted illicit drug samples

**Fig. 2:**
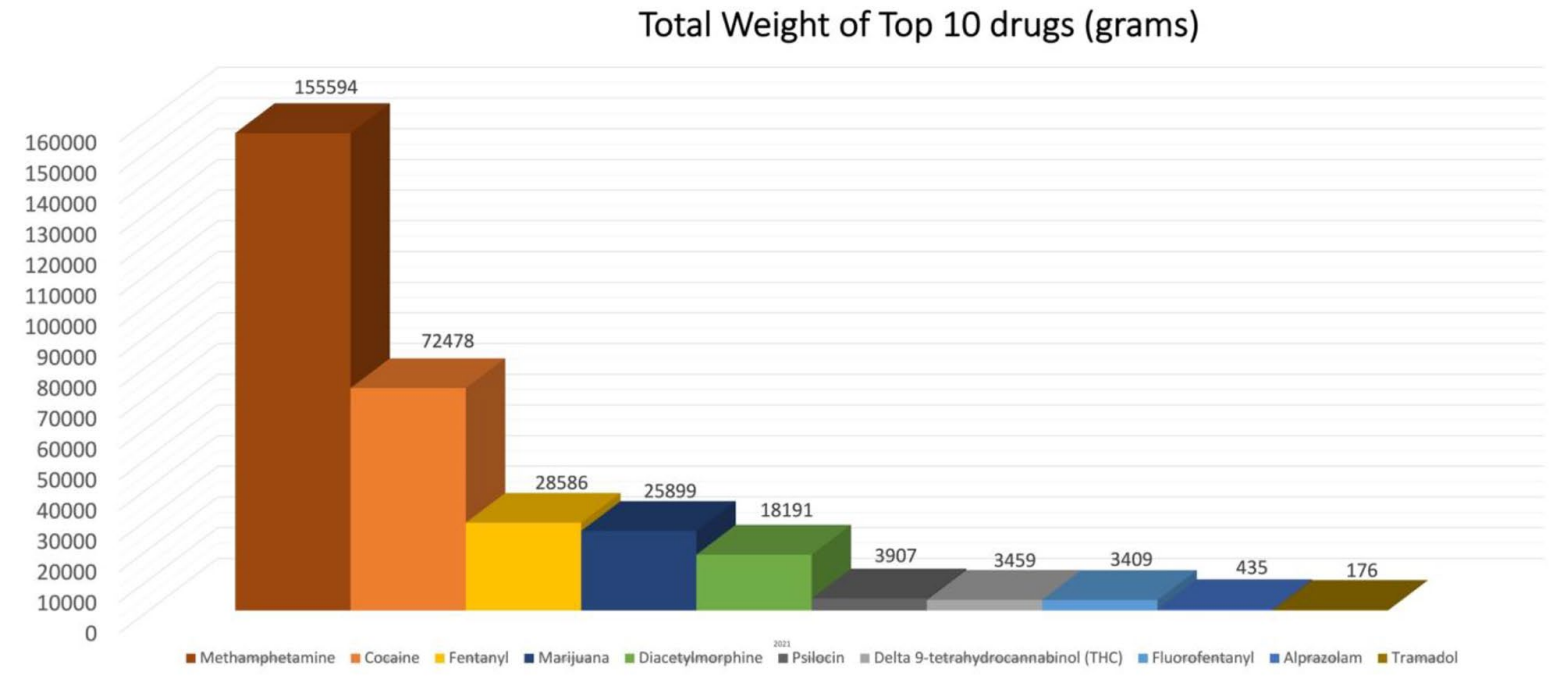
Most commonly identified illicit drugs by weight from among 4838 submitted samples

Following analysis of these samples, 39 individual drugs of abuse were identified. The most commonly identified substances were methamphetamine (38.7%), fentanyl (20.8%), diacetylmorphine (heroin) (10.2%), codeine (5.8%) and alprazolam (4.3%). Figure 1 details the relative percentages of each individual substance identified and Fig. 2 details the ten most common substances by weight.

**Fig. 3:**
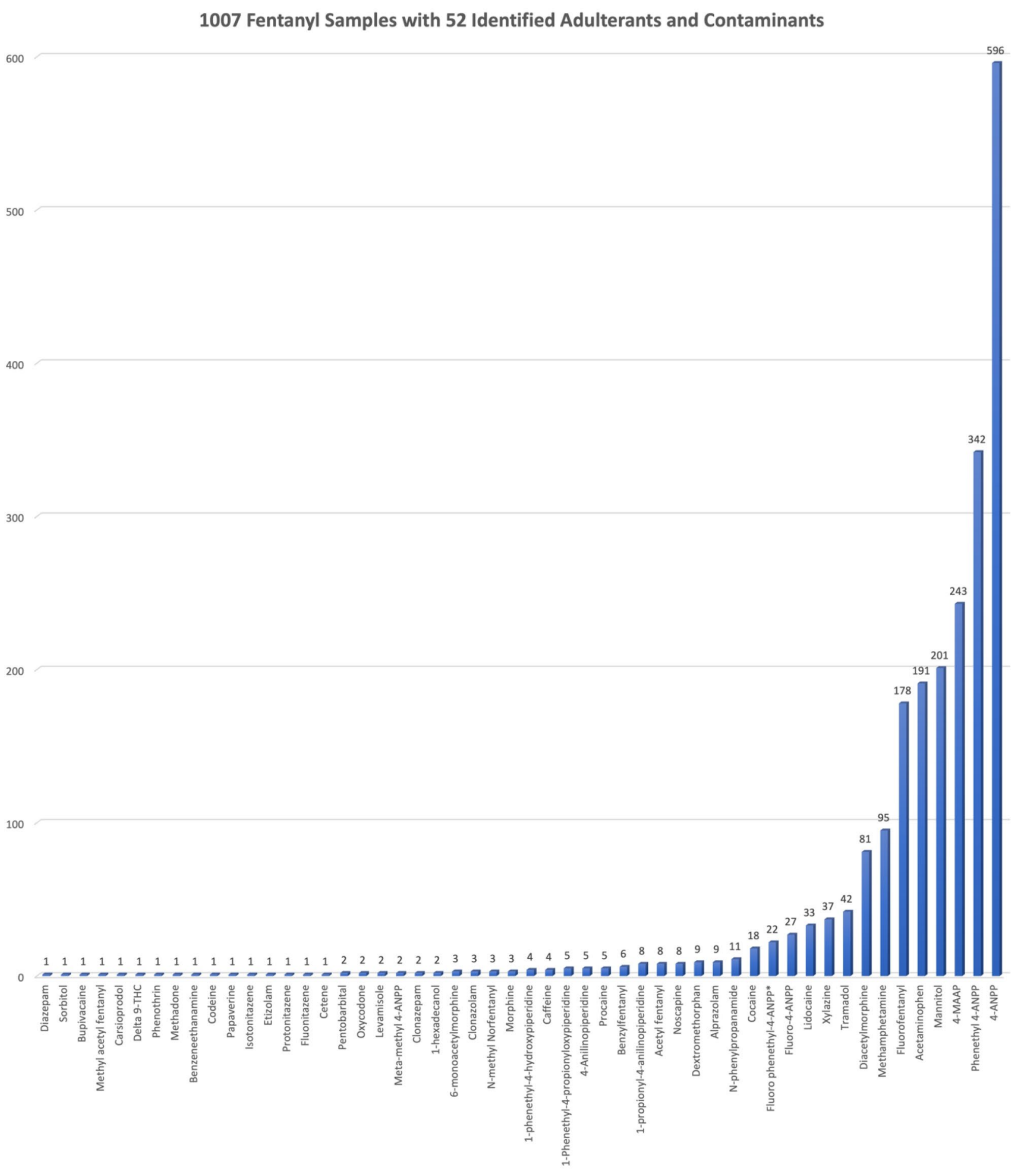
52 unique adulterants and contaminants identified within 1007 confirmed and analyzed fentanyl samples

Of the 4838 total samples tested, 1007 (20.8%) were confirmed to contain fentanyl. Qualitative analysis via FTIR and/or GC-MS of these fentanyl samples revealed the presence of 52 unique adulterants (Fig. 3). The most common included 4-methylaminoantipyrine (4-MAAP) (10.9%), mannitol (9%), acetaminophen (8.5%), methamphetamine (4.2%), diacetylmorphine (3.6%), tramadol (1.9%), and xylazine (1.7%). Various fentanyl analogues were also identified, namely fluorofentanyl, acetylfentanyl, benzylfentanyl, and methyl acetyl fentanyl. Several other opioids were detected including diacetylmorphine (heroin), morphine, 6-monoacetylmorphine, oxycodone, codeine, methadone, fluonitazene, protonitazene, and isotonitazene. Table 1 details the complete list of opioid adulterants found.

**Table 1:** Pharmacologically active opioids identified within 1007 samples of illicit fentanyl

Pharmacologically Active Opioids		
Fentanyl Analogues	Synthetic (Benzimidazole) Opioids	Non-Fentanyl Opioids
Fluorofentanyl	Fluonitazene	Diacetylmorphine
Acetyl fentanyl	Protonitazene	Tramadol
Benzylfentanyl	Isotonitazene	Morphine
Methyl acetyl fentanyl		6-Monoacetylmorphine
		Oxycodone
		Codeine
		Methadone
		Noscapine

A large number of pharmaceutical non-opioid adulterants were detected. These included acetaminophen, dextromethorphan, methamphetamine, xylazine, lidocaine, cocaine, alprazolam, procaine, caffeine, clonazepam, levamisole, phenobarbital, etizolam, delta 9-tetrahydrocannabinol, carisoprodol, bupivacaine, and diazepam. Table 2 details the complete list of pharmaceutical non-opioid adulterants. The diluents mannitol and sorbitol were also detected.

**Table 2:** Pharmacologically distinct non-opioid adulterants identified within 1007 samples of illicit fentanyl

Non-Opioid Adulterants			
Stimulants	Sedative-Hypnotics	Anesthetics	Other
Methamphetamine	Xylazine	Lidocaine	Acetaminophen (Analgesic, antipyretic)
Cocaine	Alprazolam	Procaine	Dextromethorphan
Caffeine	Clonazolam	Bupivacaine	Delta-9-tetrahydrocannabinol (Cannabinoid)
Benzeneethanamine (amphetamine analogue)	Clonazepam		4-methylaminoantipyrine (Metamizole metabolite, analgesic)
	Phenobarbital		Papaverine (Phosphodiesterase inhibitor)
	Etizolam		Levamisole (Anthelminthic)
	Carisoprodol		Mannitol, sorbitol (Diluents)
	Diazepam		

In terms of contaminants, several fentanyl precursors were noted. Among those precursors, 4-anilino-*N*-phenethylpiperidine (4-ANPP) and phenethyl-4-anilino-N-phenethylpiperidine (phenethyl 4-ANPP) were the most common. Other contaminants identified include 1-hexadecanol and 1-hexadecene. Table 3 highlights a complete listing of contaminants found within submitted fentanyl samples.

**Table 3:** Contaminants identified within 1007 samples of illicit fentanyl

Fentanyl Precursors	Others
4-Phenyl]amino-1-phenethylpiperidine (4-ANPP)	Phenothrin (Pyrethroid)
4-anilino-N-phenethylpiperidin (Phenethyl 4-ANPP)	1-hexadecene, 1-hexadecanol (surfactants)
Fluoro Phenethyl-4-ANPP	Phenylpropanamide (fentanyl degradation product)
Fluoro-4-ANPP	
Meta-methyl 4-ANPP	
4-anilino-N-phenethylpiperidin (Phenethyl 4-ANPP)	
1-Propionyl-4-Anilinopiperidine	
4-Anilinopiperidine	
1-Phenethyl-4-Propionyloxypiperidine	
1-Phenethyl-4-Hydroxypiperidine	
4 N-Methyl Norfentanyl	

## Discussion

San Diego is an epicenter for opioid trafficking given its large population and proximity to the US-Mexico border. This results in more opioid seizures than at any other domestic port of entry. In 2022, more than 2300 kg of fentanyl were seized by San Diego-based law enforcement officers, accounting for nearly 60% of all trafficked fentanyl nationwide [15, 16]. Given this proximity to upstream trafficking, it is possible that the drug supply in San Diego and the surrounding region is likely to be closer to the original trafficked product, containing fewer adulterants and contaminants than would be seen in locations distant from the entry point.

Notably, several opioid adulterants were identified, of which diacetylmorphine (heroin) was the most common. This is consistent with the Drug Enforcement Agency’s Fentanyl Signature Profiling Program, which also found diacetylmorphine (heroin) was the most common opioid adulterant in domestic illicit fentanyl supplies [17]. Fentanyl analogs fluorofentanyl, acetyl fentanyl, and benzylfentanyl have been implicated in overdoses and deaths [18–21]. The benzimidazole opioids flunitazene, protonitazine, and isotonitazene, are highly potent and have been associated with several fatalities [17].

Of the many pharmaceutical non-opioid adulterants identified, a large number were sedative-hypnotic drugs of various classes, including benzodiazepines, carisoprodol, phenobarbital, and xylazine. Co-ingestion of sedative-hypnotic agents and opioids can produce synergistic euphoric effects as well as potentially synergistic respiratory depression [22]. The combination of opioids and xylazine, or “tranq dope,” in particular, is a concerning emerging phenomenon occurring across the United States. Though the highest prevalence of xylazine overdoses and deaths has, thus far, been observed in the Northeastern United States, our data show that xylazine adulteration is present in a small percentage of recently trafficked fentanyl in our geographic region as well [23]. Acetaminophen is commonly used as a bulking agent [24].

We suspect that the presence of stimulant adulterants such as methamphetamine, cocaine, and caffeine is likely intentional. Combining opioids and stimulants in a single ingestion is known to produce a synergistic, longer-lasting euphoric effect. The combination also limits the adverse effects of either alone [25–27]. Our findings of stimulants as fentanyl adulterants may correspond with the “fourth wave” of the US drug overdose crisis, marked by escalating deaths involving cocaine and methamphetamine, alongside or without fentanyl. Despite the crisis being primarily labeled as an “opioid” or “fentanyl crisis,” recent data indicate a significant surge in overdose deaths related to psychomotor stimulants such as cocaine and methamphetamine [28]. However, it is also possible this data represents cross-contamination with other drugs during manufacturing and/or trafficking.

Though possibly adulterants of unclear value, it is more likely that the detected local anesthetics and levamisole are associated with cocaine and not added intentionally to the fentanyl. Local anesthetics are commonly used to mimic the anesthetic qualities of cocaine, whereas levamisole is reported to cause synergistic effects with cocaine [29–32].

In terms of contaminants, the most common were fentanyl precursors, 4-ANPP and phenethyl 4-ANPP. Though both possess mild mu-opioid receptor agonism, these more likely represent sequelae of poor manufacturing processes than adulterants [21, 32, 33].

The results of this study are limited by several factors. First, we analyzed convenience samples submitted by participating law enforcement agencies, which do not necessarily represent a true random sample of the drug supply in our county. Second, we cannot account for drug sample storage, processing, and handling by distributors before analysis, allowing for the possibility of contamination after seizure. Third, this study is limited in terms of its generalizability. Though our findings reflect regional trends and likely reflect the state of the supply early in the domestic trafficking pipeline, they may not necessarily be reflective of drug adulterant trends nationally. Fourth, our analysis was qualitative rather than quantitative and did not generate relative composition data. Consequently, we could not report relative concentrations of drug versus adulterants nor the proportion of pure samples. Fifth, our study conducted non-targeted analysis of unknown drug supplies, with the recognition that intentionality behind the adulterants could not be discerned due to limitations inherent in the data provided by law enforcement. We focused on characterizing the adulterants present within these samples within the constraints of the provided data. Finally, this study lacks individual case details or any outcomes data from the sample’s use.

## Conclusion

Ultimately, we feel this information is vital for public health use and harm reduction at the level of the individual consumer. Continued direct surveillance of the drug supply is necessary for the detection of potentially harmful adulterants that may pose serious threats to consumers. However, the extent to which these unique adulterants impact drug use as well as associated morbidity and mortality remains uncertain and an area of interest for further study.

## Data Availability

All relevant data sets made available by the San Diego County Sheriff’s Department Regional Crime Laboratory. In accordance with California state and San Diego local regulatory requirements the collected data sets cannot be made public due to their sensitive nature. Please email Ms. Jennifer Harmon at Jennifer.Harmon@sdsheriff.org to discuss requesting data from this study.
